# Influence of Iron Deficiency on HbA1c Levels in Pregnant Women: Comparison with Non-Pregnant Women

**DOI:** 10.3390/jcm7020034

**Published:** 2018-02-20

**Authors:** Kunihiko Hashimoto, Masafumi Koga

**Affiliations:** 1 Division of Diabetes and Endocrinology, Department of Internal Medicine, NTT West Osaka Hospital, Osaka 543-8922, Japan; kunijin@zeus.eonet.ne.jp; 2 Department of Internal Medicine, Hakuhokai Central Hospital, Amagasaki, Hyogo 661-0953, Japan

**Keywords:** HbA1c, glycated albumin, pregnancy, iron deficiency state

## Abstract

Although HbA1c is widely used as a glycemic control indicator, HbA1c is known to show falsely high levels in patients in an iron deficient state (IDS). We compared the influence of IDS on HbA1c levels between pregnant women, due to mainly an increase in demand for iron without bleeding, and non-pregnant women, due to mainly bleeding (menstruation). We studied 42 non-diabetic pregnant women (pregnant group) and 42 age-matched non-pregnant women with normal glucose tolerance (non-pregnant group). We compared HbA1c and glycated albumin (GA) levels between IDS and normal iron state (NIS) in both groups. Furthermore, we analyzed the correlation between indicators of glycemic control and iron-related parameters [mean corpuscular hemoglobin, serum transferrin saturation (%Tf), and serum ferritin] in both groups. Compared with non-pregnant women, pregnant women had significantly lower %Tf and serum ferritin levels and significantly higher morbidity of IDS. HbA1c, but not GA, had significantly higher levels in pregnant women with IDS compared with NIS; however, HbA1c in non-pregnant women showed no significant difference for both IDS and NIS. In pregnant women, significant negative correlations were observed between HbA1c and iron-related parameters. In non-pregnant women, negative correlations were observed between HbA1c and these parameters, but they were not significant. No significant correlations were observed between GA and iron-related parameters in both groups. HbA1c levels in pregnant women were found to be largely affected by iron deficiency compared with non-pregnant women. For this reason, GA, which is not affected by iron deficiency, is desirable for use in the assessment of glycemic control during pregnancy.

## 1. Introduction

Currently, HbA1c is widely used for the target value of glycemic control or for diagnosis of diabetes mellitus, the gold standard indicator of glycemic control [1]. However, HbA1c is influenced by a variety of physical factors and it may not reflect glycemic control accurately. Such factors include shortened erythrocyte lifespan, such as by bleeding, hemolytic anemia, renal anemia, extended erythrocyte lifespan, such as by vitamin B12 deficiency, or variant hemoglobin [2,3,4,5].

Coban and colleagues reported that HbA1c showed falsely high levels in patients with iron deficiency anemia [6]. However, subsequent studies have documented that HbA1c in IDA or IDS patients showed slightly high levels [7,8,9,10]. We demonstrated that there was a significant negative correlation between HbA1c and iron-related parameters in premenopausal women with not only iron deficiency anemia but also iron deficiency state (IDS) without anemia [11] and HbA1c in IDS women exhibited significantly higher levels compared with the normal iron state (NIS) [7]. On the other hand, glycated albumin (GA) was not affected by iron deficiency [7]. Among non-menopausal women, HbA1c in women with iron deficiency anemia simply showed slightly higher levels compared to women without iron deficiency [7].

HbA1c is known to increase in late pregnancy [12,13]. We reported that HbA1c increases significantly as iron deficiency proceeds during pregnancy in non-diabetic pregnant women, pregnant women with gestational diabetes, and pregnant women with overt diabetes [14,15]. Furthermore, we documented that IDS occurs more often in late pregnancy as demand for iron increases and other factors (inflammation etc.). HbA1c showed falsely higher levels than HbA1c estimated mean blood glucose obtained by continuous glucose monitoring due to IDS [16]. In addition, the extent of the elevated HbA1c level associated with IDS in late pregnancy was outstanding compared with non-menopausal women. However, GA was not affected by IDS even in pregnant women [14]. Although it is known that hemodilution occurred in pregnancy, both biomarkers (HbA1c and GA) are not influenced because these biomarkers express the ratio of glycated protein to whole protein.

In this study, we analyzed the varying influence of HbA1c by iron deficiency in both groups by examining and comparing the relationship of IDS and HbA1c in pregnant and non-pregnant women.

## 2. Patient and Methods

### 2.1. Patients

We studied 42 pregnant Japanese women at gestational weeks 21–36. All subjects had visited Aizenbashi Hospital and randomly measured plasma glucose levels were <100 mg/dL (pregnant group). We also studied 42 age-matched control women who visited the Health Care Center at Kinki Central Hospital for a health examination (non-pregnant group). Fasting blood was collected from both groups. All controls had a 75 g oral glucose tolerance test and their glucose tolerance status was diagnosed as normal according to the World Health Organization criteria [17]. Patients suffering from hepatic or renal diseases and/or subjects with high C reactive protein levels were excluded.

IDS was defined as having a ferritin level below 15 ng/mL. We compared HbA1c and GA between IDS and NIS in both groups. Furthermore, we analyzed the correlation between indicators of glycemic control (HbA1c and GA) and iron-related parameters [mean corpuscular hemoglobin (MCH), serum transferrin saturation (%Tf), and serum ferritin] in both groups.

The reported investigations have been carried out in accordance with the principles of the Declaration of Helsinki, as revised in 2000. The institutional review board approved this study, and all patients gave their written informed consent.

### 2.2. Laboratory Methods

HbA1c, expressed as the National Glycohemoglobin Standardization Program (NGSP) value [18], was measured by high-performance liquid chromatography using ADAMS-A1c HA-8160 (Arkray Inc., Kyoto, Japan) [19]. GA was determined by an enzymatic method using albumin- specific proteinase, ketoamine oxidase and albumin assay reagent (Lucica GA-L; Asahi Kasei Pharma Co., Tokyo, Japan) [20], with the use of a Hitachi 7600 auto-analyzer (Hitachi Instruments Service Co., Tokyo, Japan). The blood cell counts, hematocrit (Ht), hemoglobin (Hb), mean corpuscular volume (MCV) and MCH were measured by an automated hematology system (XT–2000i; Sysmex Co., Kobe, Japan). The serum iron and unsaturated iron-binding capacity (UIBC) were determined by a calorimetric method using a Hitachi 7700 auto-analyzer (Hitachi Instruments Service Co., Tokyo, Japan). The serum ferritin concentrations were measured by the chemi- luminescent immunoassay (CLIA) method (ADVIA Centaur: Siemens Medical Solutions Diagnostics Co., Tokyo, Japan). The total iron-binding capacity (TIBC) and %Tf were calculated by adding UIBC to the serum iron and dividing the serum iron by TIBC, respectively.

### 2.3.Statistical Analyses

Data are shown as the mean ± SD for the continuous variables. An unpaired t-test was used to estimate the level of significance of the differences by mean. To evaluate the relationship between HbA1c levels and different variables, single linear univariate regression analyses were performed. The StatView computer program (Version 5.0 for Windows; Abacus Concepts, Berkeley, CA, USA) was used for all statistical analyses. A *p* value of <0.05 was considered statistically significant.

## 3. Results

The clinical characteristics of the pregnant women and non-pregnant women are shown in Table 1. The gestational weeks of the pregnant women were 27.5 ± 5.1 weeks in mid- to late- pregnancy. The red blood cell count, Hb and Ht in pregnant women, exhibited significantly lower levels compared with non-pregnant women. Furthermore, both the %Tf and serum ferritin in pregnant women showed significantly lower levels compared with non-pregnant women. The incidence of IDS observed in pregnant women was 64.3%, which was significantly higher compared with 34.1% in non-pregnant women (*p* = 0.004). HbA1c in pregnant women was significantly lower than in non-pregnant women (4.88 ± 0.33% vs. 5.14 ± 0.22%, *p* < 0.001), and GA in pregnant women was significantly lower than in non-pregnant women (13.9 ± 1.0% vs. 14.5 ± 1.2%, *p* = 0.013).

RBC, red blood cell; MCH, mean corpuscular hemoglobin; Tf, transferrin; IDS, iron deficient state.

**Figure 1:**
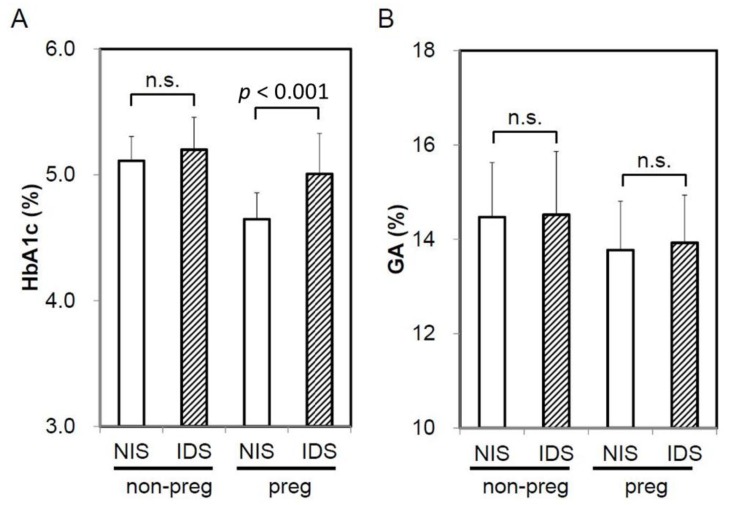
Influence of iron deficiency on HbA1c and GA levels in non-pregnant and pregnant women. Comparison of HbA1c (**A**) and GA (**B**) between IDS and NIS in non-pregnant women and pregnant women were shown. NIS, normal iron state; IDS, iron deficient state; non-preg, non-pregnant women; preg, pregnant women; n.s., not significant.

**Figure 2:**
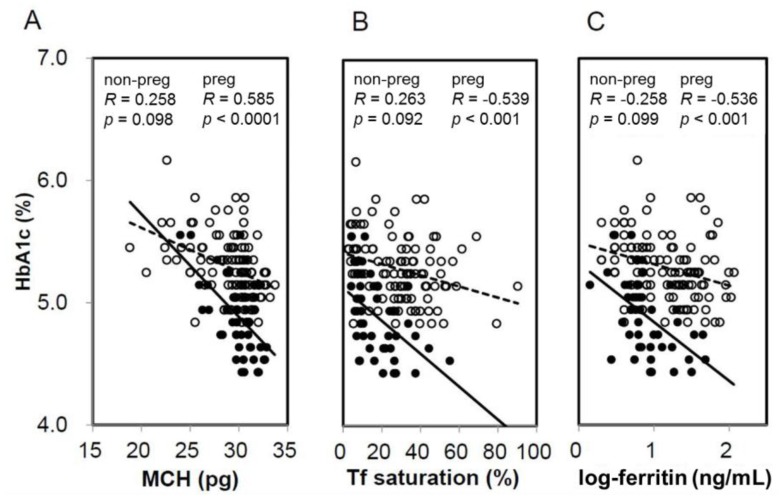
Correlation between iron-related parameters and HbA1c in non-pregnant and pregnant women. Correlation was shown between MCH (**A**), transferrin (Tf) saturation (**B**), and logarithm-transformed ferritin (**C**) and HbA1c in non-pregnant (open circles, dotted line) and pregnant women (closed circles, straight line). non-preg, non-pregnant women; preg, pregnant women.

When HbA1c and GA were compared based on the presence or absence of IDS, HbA1c in pregnant women with IDS had significantly higher levels compared with NIS. Meanwhile, HbA1c in non-pregnant women exhibited no significant difference between IDS and NIS (Figure 1A). On the other hand, GA showed no significant difference between the presence and absence of IDS in both pregnant women and non-pregnant women (Figure 1B). In addition, a significant negative correlation was observed between HbA1c and MCH (*R* = −0.585, *p* < 0.0001), %Tf (*R* = −0.536, *p* < 0.001), and ferritin (*R* = −0.539, *p* < 0.001) in pregnant women (Figure 2). Meanwhile, a negative correlation trend was observed between HbA1c and MCH (*R* = −0.258, *p* = 0.098), %Tf (*R* = 0.263, *p* = 0.092), and ferritin (*R* = 0.058, *p* = 0.726), but it was not significant in non-pregnant women. There was no significant correlation between GA and iron related parameters in pregnant and non-pregnant women (preg-MCH: *R* = 0.160, *p* = 0.313; %Tf: *R* = −0.174, *p* = 0.434; ferritin: *R* = −0.084, *p* = 0.595; non-preg-MCH: *R* = 0.124, *p* = 0.434; %Tf: *R* = 0.058, *p* = 0.726; ferritin: *R* = 0.051, *p* = 0.749).

## 4. Discussion

Both HbA1c and GA levels in pregnant women were significantly lower than in non-pregnant women. The levels were particularly lower than non-pregnant women, although HbA1c in pregnant women can reach high levels under the influence of IDS. The phenomenon in which HbA1c and GA showed lower levels was in line with the reports [13] presenting that plasma glucose in pregnant women showed lower levels compared with non-pregnant women. Although the reason why the plasma glucose level becomes lower during pregnancy is unknown, a mechanism that prevents hyperglycemia might exist even in non-diabetic women because hyperglycemia can result in perinatal complications.

The %Tf and serum ferritin in pregnant women were at significantly lower levels compared with non-pregnant women, and IDS morbidity was also at a significantly higher rate. In addition, in non-pregnant women, no significant difference in HbA1c levels was observed between IDS and NIS. Meanwhile, HbA1c levels in IDS were significantly higher compared with NIS in pregnant women. In other words, it was demonstrated that, as the extent of the elevated HbA1c levels in non-pregnant women with IDS remained only slight, HbA1c in pregnant women with IDS showed higher outstanding levels.

IDS or IDA in non-pregnant women develops primarily from bleeding associated with menstruation and an iron-deficient intake corresponding to the bleeding. On the other hand, women experience amenorrhea during pregnancy, with no bleeding. Demand for iron increases with fetal development, and when iron intake cannot meet the demand, IDS or IDA occurs. Coban and colleagues reported that HbA1c in patients with IDA presented outstanding high levels [6]. However, subsequent studies have documented that HbA1c in IDA or IDS patients showed slightly high levels [7,8,9,10]. In any case, such levels would be due to IDS with menstrual bleeding, where HbA1c in premenopausal women with IDS remained at slightly higher levels. After bleeding, the synthesis of erythrocytes increases and the erythrocyte lifespan shortens, leading to lower levels of HbA1c. If it subsequently becomes IDS, the synthesis of erythrocytes would be delayed, and HbA1c levels would increase. Thus, in patients with IDS and menstrual bleeding, the extent of HbA1c levels appears to remain slightly higher due to the coexistence of the HbA1c decrease associated with bleeding and the HbA1c increase associated with IDS.

The mechanism of HbA1c elevation in patients with IDA is currently unknown. It has been reported that the erythrocyte lifespan in patients with IDA was not extended and remained normal or slightly shortened [21,22]. However, most of the reported patients with IDA had underlying gastrointestinal bleeding diseases. Therefore, affected by the shortened erythrocyte lifespan associated with bleeding, the assessment of the erythrocyte lifespan in patients with IDA themselves seems to not have been achieved. Erythrocyte creatine, which reflects the erythrocyte lifespan, resulted in significantly higher levels during menstruation for women in their 10s to 40s compared to men of the same age. However, difference by sex was not shown in <10-year-old and ≥50-year-old patients [23]. This result is considered to indicate that the erythrocyte lifespan is shortened due to bleeding for women with menstruation. For patients with IDA without bleeding, an extended erythrocyte lifespan might result in high levels of HbA1c. This issue should be considered in the future.

As a matter of course, women experience amenorrhea during pregnancy, with no bleeding. However, demand for iron increases as the fetus grows and IDA may occur and HbA1c levels may increase, unless iron intake can meet the demand. It is assumed that the number of pregnant women who experience IDS would increase in late pregnancy, and HbA1c levels would be elevated from mid-pregnancy to late pregnancy because iron demand increases as the gestational weeks continue. That is to say, it seems that an influence on HbA1c is markedly observed compared to IDS associated with bleeding because the influence of HbA1c on pure IDS is observed during pregnancy.

In IDS patients without bleeding, HbA1c markedly results in high levels. Therefore, under these conditions, estimation of glycemic control based on HbA1c levels may lead to a risk of misjudging. It is desirable make assessments based on GA in these diseases because GA is not affected by IDS [14,15]. Traditionally, compared with HbA1c, GA was proposed to be used as a glycemic control indicator during pregnancy [15,24] because GA reflects glycemic control over a short period. Recently, no significant difference has been observed between HbA1c in late pregnancy and perinatal complications, and GA in late pregnancy has been reported to exhibit a significant difference [24,25]. HbA1c does not serve as a predictive factor of perinatal complications, probably because HbA1c during pregnancy shows falsely high levels under the influence of IDS. However, GA appears to be a significant related factor to perinatal complications because GA accurately reflects glycemic control without the influence of IDS. Therefore, glycemic control during pregnancy should be assessed using GA.

IDS without bleeding includes iron malabsorption after gastrectomy, iron-deficient intake due to an unbalanced diet and going on a diet, excluding demand enhancement such as pregnancy. In these diseases, HbA1c may result in outstanding falsely high levels due to IDS, thus caution should be exercised when assessing glycemic control with HbA1c.

This study has some limitations. First, the sample size was not large. Second, we did not recruit diabetic pregnant women in this study It is necessary to compare diabetic pregnant women with diabetic non-pregnant women in near future. Third, serum ferritin levels in pregnant women are decreased due to hemodilution. However, diagnosis of IDA in pregnant women is usually performed using the serum ferritin [26]. Fourth, the cases used for the pregnant and non-pregnant women were from different institutions. Therefore, this study was preliminary work to guide a more detailed, in depth follow-up, and it is necessary to confirm the findings of this study using a large sample from multiple institutions in the future.

In conclusion, we demonstrated that HbA1c in pregnant women is greatly affected by iron deficiency compared with non-pregnant women. For this reason, GA, which is not affected by iron deficiency, is appropriate to use as indicator of glycemic control during pregnancy.

## Figures and Tables

**Table 1:** Clinical characteristics of study subjects.

	Non-Pregnant Women	Pregnant Women	*P* Value
*n*	42	42	-
Age (years)	31.6 ± 4.0	29.9 ± 5.9	0.120
Pregnancy (week)	-	27.5 ± 5.1	-
RBC (×10^4^/μL)	429 ± 26	361 ± 21	<0.0001
Hb (g/dL)	12.6 ± 1.3	10.8 ± 0.7	<0.0001
Ht (%)	37.0 ± 3.3	33.8 ± 2.0	<0.0001
MCH (pg)	29.4 ± 2.7	30.0 ± 2.1	0.274
Tf saturation (%)	31.2 ± 19.0	18.4 ± 11.8	<0.001
Ferritin (ng/mL)	29.6 ± 26.0	11.4 ± 11.1	<0.0001
IDS (%)	14 (34.1)	27 (64.3)	0.004
HbA1c (%)	5.14 ± 0.22	4.88 ± 0.33	<0.0001
Glycated albumin (%)	14.5 ± 1.2	13.9 ± 1.0	0.013
